# Coping with light pollution in urban environments: Patterns and challenges

**DOI:** 10.1016/j.isci.2024.109244

**Published:** 2024-02-16

**Authors:** Ulrika Candolin

**Affiliations:** 1 Organismal and Evolutionary Biology Research Programme, University of Helsinki, Helsinki, Finland

**Keywords:** Classification Description, Nature conservation, Ecology, Evolutionary biology

## Abstract

Artificial light at night is a growing environmental problem that is especially pronounced in urban environments. Yet, impacts on urban wildlife have received scant attention and patterns and consequences are largely unknown. Here, I present a conceptual framework outlining the challenges species encounter when exposed to urban light pollution and how they may respond through plastic adjustments and genetic adaptation. Light pollution interferes with biological rhythms, influences behaviors, fragments habitats, and alters predation risk and resource abundance, which changes the diversity and spatiotemporal distribution of species and, hence, the structure and function of urban ecosystems. Furthermore, light pollution interacts with other urban disturbances, which can exacerbate negative effects on species. Given the rapid growth of urban areas and light pollution and the importance of healthy urban ecosystems for human wellbeing, more research is needed on the impacts of light pollution on species and the consequences for urban ecosystems.

## Graphical abstract

**Figure:**
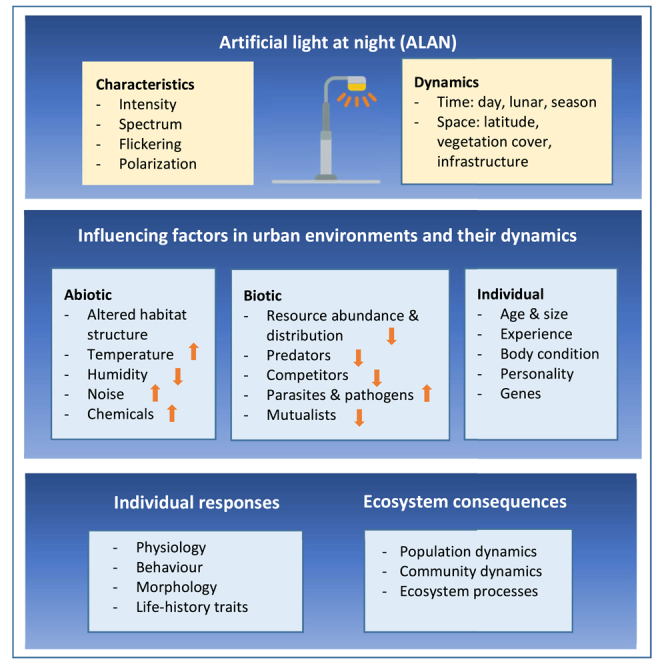

## Introduction

Light pollution, in terms of artificial light at night, ALAN, is a growing environmental problem. It alters natural cycles of light, including daily, lunar, and seasonal cycles, which regulate various processes of living organisms, from budburst in plants to behavior of animals.^1^ In addition, artificial light fragment habitats attract some species while repelling others, and alter the ability of individuals to find resources or camouflage themselves from predators.^2^ Thus, light pollution disrupts vital biological processes and alters the behavior, abundance, and spatiotemporal distribution of species. This, in turn, can influence species interactions and the composition and function of species communities. Such disruptions can have far reaching consequences for ecosystems, from reduced pollination success to the facilitated spread of pathogens and pests.^3^^,^^4^

The impact of light pollution on species and ecosystems is currently gaining increasing attention, given the accelerated growth of the use of ALAN.^5^ However, research has mainly focused on organisms in their natural environment or under laboratory or husbandry conditions, while less is known about the impact on wildlife in urban environments. Yet, light pollution is most severe in cities and towns, and urban land is rapidly expanding, expected to increase by 2–6-fold over the 21st century.^6^ Thus, more and more organisms will be exposed to strong ALAN. Urban environments are also exposed to a range of other stressors and disturbances, such as elevated temperature, higher noise levels, chemical pollution, and altered habitat structure. These changes can interact with light pollution and amplify its negative effects on species. Yet, some species have been able to adjust to light conditions in cities, and some even thrive under the new conditions, such as rats and pigeons. However, many are struggling and the degree to which they will be able to adjust and adapt is poorly known.

This lack of attention to the impacts of artificial light on urban wildlife is surprising considering the attention that has been given to impacts on humans. Much evidence exists on negative effects on human health and performance, such as disrupted sleep, increased stress levels, impaired repair and recovery of physiological functions, depression, reduced performance of immune system, and heightened risk of developing cancers and cardiovascular disease.^7^ Similar effects are likely to occur in animals inhabiting urban environments.^8^

**Figure 1:**
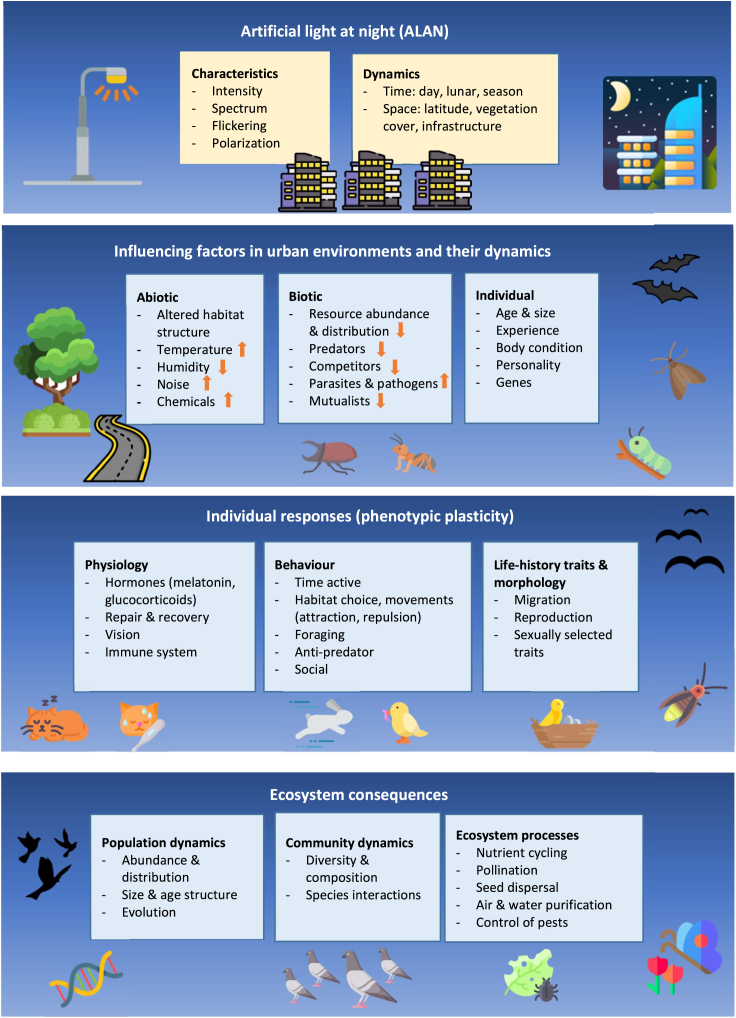
The various factors that can influence the impact of artificial light at night on organisms in urban environments, how the individuals may respond to the light, and the influence their responses may have on the components of ecosystems Common differences between urban and natural environments in abiotic and biotic factors are indicated with colored arrows.

Given the importance of species composition and biodiversity for the function and resilience of urban ecosystems, we need a better understanding of the factors that influence species in these ecosystems. Here, I discuss the challenges that species encounter when attempting to cope with urban light pollution, how the challenges can be exacerbated by interactions with other urban disturbances, and how species may respond in terms of plastic adjustments and genetic adaptation. I examine the evidence for adaptive and maladaptive responses to light pollution, and the ecological consequences the responses may have. I end with outlining gaps in our knowledge and how filling them could advance the research field. Overall, the aim is to present a conceptual framework for how animals respond to light pollution in urban environments, the patterns and underlying mechanisms, and possible ecological consequences (Figure 1). Hopefully, this will inspire more research into this important topic, considering that little attention has so far been paid to the impact of light pollution on urban wildlife, although urban areas and light pollution are rapidly growing around the world.

## Artificial light at night in urban environments

The intensity of artificial light in urban environments varies both spatially and temporally. It is usually strongest in city centers and decreases in intensity toward suburban and rural areas, where the density of roads and buildings is less dense (although local constructions, such as harbors and industrial parks, can strongly light up an area).^9^^,^^10^ Skyglow, when some of the light that radiates upwards from an urban area is diverted back to earth through scattering, can reach far beyond the urban area.^11^ Light intensity within urban areas varies in turn depending on infrastructure, usually being brightest along busy streets, in parking lots, at stadiums, and around shopping malls.^12^ The intensity is generally highest in the evening after darkness has fallen, decreases when some lights are turned off, but may increase again toward dawn, with seasonal and latitudinal differences in timing.^1^^,^^13^

Similarly, the spectral character of the light varies within urban areas. Yellow or white light is common along streets and paths, while multiple colors are often used in digital billboards and signs. The current conversion to light emitting diodes, LEDs, to save energy and costs, is altering the spectrum of light, from the yellow-orange light of traditional bulbs, such as sodium bulbs, to the white light of broad spectrum LEDs.^14^ The stronger emission of LEDs in the blue range stimulates melanopsin-expressing ganglion cells and interferes with the production of melatonin, which in turn can influence circadian rhythms and sleep patterns and thereby traits that influence survival and reproduction.^8^^,^^15^^,^^16^ Blue light also enhances the positive phototactic response of many insects to artificial light, which causes them to be trapped around the light.^17^ Given that light intensities of LEDs usually are stronger than that of older lamps, LED installations have worsened the light pollution problem.^14^

Other characteristics of artificial light that can influence the performance of animals is flickering. It can cause stress to animals and alter their behavior and health.^18^ It can also change the polarization of light reflected from artificial surfaces, such as asphalt, glass surfaces, and dark-colored cars, which can disrupt the orientation of animals and hamper the ability of aquatic insect to judge suitable oviposition sites.^19^

## Expected impacts of light pollution on urban animals

The stronger artificial light in urban environments compared to natural environments, combined with altered habitat structure, is likely to amplify the influence of artificial light on the physiology and behavior of animals. Impermeable surfaces, limited green areas, less water bodies, and restriction of movement by buildings and streets may reduce the ability of animals to find hiding places from the artificial light. The high density of humans and the concentration of their activities to daytime may in turn cause animals to increase their activity during the night, further increasing their exposure to artificial night light. In addition, the abundance of other stressor, such as traffic, noise, higher temperature, and larger fluctuations (urban heat island) may stress animals and increase their sensitivity to artificial light. Variation in light conditions within urban areas may in turn increase animal densities in areas with less artificial light and limit movements and gene flow.

Exposure to ALAN can influence the fitness of animals both directly and indirectly. Common direct effects are altered habitat choice and activity time. For instance, many insects are attracted to artificial light, which can result in them being killed upon contact, or exhausted when flying around the light source. Light at night can also alter the ability of animals to find prey, their visibility to predators, or their ability to attract mates.^20^^,^^21^

Indirect effects, including physiological changes that influence behavior, and effects through interactions with other affected species, are likely to be common although little investigated in an urban context.^22^ For instance, artificial light is likely to suppress melatonin concentration and thereby influence behaviors related to fitness.^8^ It can also induce stress and increase the levels of glucocorticoid hormones, which in turn can alter activity and metabolism, with further consequences for growth, survival, and reproductive success.^23^

Altered seasonal cycles of light because of light pollution can in turn change the timing of activities, such as migration, molt, hibernation, and the onset or termination of reproduction.^1^ Such changes can have knock-on effects on species interactions. For instance, earlier budburst of plants and the appearance of insect prey can lead to mistimed arrival of migratory birds in relation to food abundance, resulting in lower reproductive success. Such mismatches among trophic levels have been recorded in relation to climate change,^24^ but could be common also under light pollution.

Indirect effects through species interactions are likely to be common, as changes in the abundance or traits of one species can cause effects that ripple through the species interaction web. Thus, also species not directly affected by the artificial light can be affected.^25^^,^^26^ For instance, the attraction of insects to artificial light attracts their predators, such as bats and birds,^27^ and changes in the population dynamics of the predators can, in turn, influence their competitors or parasites, resulting in complex sequences of effects.

The molecular mechanisms behind the effects of artificial light on physiology and behavior have recently gained some attention. Impacts through clock-genes and melatonin-related genes have been implicated, but effects also arise through genes unrelated to these.^28^^,^^29^ Exposure to light at night can in turn have carry-over effects on daytime activities, although the underlying mechanisms are still poorly understood.^30^^,^^31^

Light pollution that alters the movements of individuals between rural and urban areas can influence gene flow and the genetic composition of urban populations. Similarly, variation in artificial light levels within urban areas can restrict movements and result in sub-populations that differ in genetic composition.^32^ For instance, linearly lit features, such as roads and footpaths, can form barriers to movement and subdivide populations. Increases in population densities in smaller dark refuges can again increase tensions and aggression, both within and between species.^33^ The opposite may occur in strongly lit areas, which are avoided by many species and tolerated by only a few.

Thus, light pollution can influence the fitness of individuals through effects on their growth, health, survival, movements, and reproduction. This can, in turn, alter the composition of urban communities and ecological processes, such as nutrient cycling and primary production, the transfer of energy to higher trophic levels, pollination, seed dispersal, the purification of water and air, and the suppression of pest species.^34^ Such effects of artificial light are poorly known for urban ecosystems, but could be stronger than in natural ecosystems considering the strength of artificial light. A critical question then becomes, what determines whether a species is able to adjust and/or adapt to urban light pollution. In the following paragraphs, I will discuss such influencing factors.

## Possibility of adjusting and adapting to urban light pollution

To survive and reproduce in urban environments where natural light conditions are disrupted by artificial light, animals need to adjust their physiology and behaviors to the new conditions, such as the timing of their activities and habitat choice. Species may search out darker refuges when night falls and artificial lights are lit, and night active species may change their peak activity to later in the night, if artificial light intensities are lower than. The changes may be in the form of plastic adjustments, i.e., within-individual changes, or they may require genetic adaptation through selection across generations.^35^ Plastic adjustments can be more or less immediate, depending on the need for developmental changes, while genetic adaptation is a much slower process.

A combination of rapid plastic adjustments and longer term genetic adaptation may be most efficient for securing persistence. However, this may not always be possible. The ability to plastically adjust depends on earlier evolved reaction norms and, hence, on earlier encountered environmental conditions and selection pressures.^36^ Given that artificial light is a relatively new phenomenon, most species may lack adaptive reaction norms. Some degree of plasticity may, however, exist, as natural night light conditions vary depending on moonlight, cloudiness, and habitat structure.^37^ Moreover, species that have experienced large seasonal variation in day length could be more plastic than species from more stable light conditions and more likely to successfully adjust to light pollution. Similarly, migrating species that move between habitats and latitudes may be more plastic than sedentary species.

However, urban environments differ in many respects from natural environments, as earlier discussed. This may increase the difficulty of plastically adjusting to light pollution. For instance, natural hiding places may be lacking, predators may employ unfamiliar hunting strategies, and the clumped distribution of resources may require a higher level of movements than in the natural environment. Such impediments may magnify the impact of light pollution on species.

The likelihood of genetic adaptation to light pollution depends, in turn, on the generation time of the species, the presence of genetic variation in the direction of selection, and the strength of selection.^38^ Species with short generation time and large population size are most likely to genetically adapt to light pollution. Given that population sizes are often smaller in urban areas and gene flow restricted, the potential for genetic adaptation may be more restricted than in the natural habitat. On the other hand, the selection pressure may be stronger given the higher light intensity and limited ability to escape the light.

The time the species has been exposed to the artificial light is likely to influence the likelihood of adaptive responses; species residing in older urban areas are more likely to have evolved adaptive reaction norms than species in more recently urbanized areas. Given that the widespread adoption of artificial light did not begin until the mid-20^th^ century, it is no surprise that the species faring best in urban environments are species with short generation time and large reproductive output.^35^

Transgenerational, plastic effects, when the parental, and possible grand-parental, generation influences the traits of the next generation through non-genetic effects, could promote persistence, but effects in relation to light pollution has so far received little attention but see.^39^ The number of generations transgenerational effects persist are also poorly known, and the effect could hamper adjustment if light conditions change from the parental to the offspring generation.^40^ Cultural transmission of novel behaviors, such as learning to feed on insects under street lamps by observing the behaviors of others, could occur in species with more developed cognitive functions,^41^ but has not been documented in relation to light pollution.

## Evidence for animals adjusting or adapting to urban light pollution

A growing number of studies document plastic adjustments to urban environments, and some even find indications of genetic adaptation reviewed in,^42^^,^^43^^,^^44^ although robust evidence for adaptive evolution is limited.^45^ The impact of light pollution as a driving force in adaptation to urban environments is, however, seldom considered, and to separate out the effects of artificial light from that of other disturbances is challenging.

Traits that are often favored in urban environments are boldness and exploratory behavior, sophisticated cognitive skills, and lower stress responses.^46^ Whether the same traits are favored by light pollution is unknown and likely to be species-specific. Moreover, traits favored by light pollution could conflict with other demands placed by the urban environments. For instance, while a clumped distribution of resources may favor high mobility, light pollution may restrict movements by increasing visibility to predators.

Many urban dwellers appear not to suffer from light pollution, such as rats, pigeons, squirrels, and great tits. Some even benefit from the light, such as those that prey on insects trapped around street lights.^47^ For instance, the local expansion of the bat *Pipistrellus pipistrellus* in Switzerland has been hypothesized to be promoted by the concentration of insects under street lamps.^27^ On the other hand, the bats may become easier prey for urban predators, such as cats, or be hit by vehicles passing under the streetlight.^48^ In general, most night active species appear less well adapted to artificial light; birds migrating at night are colliding with lit buildings,^49^ insects are trapped around street lights,^50^ newly hatched sea turtles crawl toward cities instead of the ocean,^51^ and glow-worm females fail to attract males.^52^^,^^53^

The ability of animals to adjust and possibly adapt to light pollution depends also on how other species are affected, i.e., the species they are connected to, directly or indirectly.^54^ This can result in complex feedback loops and time lags that make it difficult to predict the ultimate effect of light pollution on communities.

While the initial success in colonizing urban environments is likely to depend on exaptations and plastic adjustments, genetic changes may accrue over time.^35^ However, only a few studies have found indications of genetic adaptation to light pollution. Altermatt and Ebert^55^ showed that Ermine moths (*Yponomeuta cagnagella*) are less attracted to artificial light in cities than in rural environments. Two residential songbirds have in turn developed smaller eyes in urban-core than in urban-edge habitats, while two migrating species have not, probably because the migrating species visit multiple areas that differ in light pollution.^56^

Urban populations usually have a lower effective size and genetic diversity than populations in natural environments. This can lower their capacity to genetically adapt to light pollution while increasing the risk of genetic drift.^57^^,^^58^ Non-random colonization of urban areas, and non-random sorting within urban areas in relation to light pollution, can in turn result in genetic differentiation both between urban and rural populations and within urban areas. Such changes, whether by selection, non-random colonization and sorting, or genetic drift, could result in species differentiation, which with time could result in speciation.^59^

## Interactions with other disturbances

Urban environments differ in many respects from natural environments, in elevated noise levels, altered sound frequencies, higher temperature, more impervious surface cover and chemical pollution.^60^ These stressors and disturbances can interact additively or synergistically and alter the ultimate impact of light pollution on species.^61^ The impact can be highly species-specific and differ depending on habitat choice, activity time, and life history. Moreover, interactive effects can be nonlinear and vary with the spatiotemporal context and life-stage of the individual, as well as depend on interactions within and among species and how these vary over time and space. Such complexities make it difficult to predict the ultimate impact of light pollution on urban wildlife.

Light pollution is especially likely to interact with global warming, as both factors influence activity and the timing of life history events, such as migration and reproduction.^62^ Their combined effect could influence species interactions, such as the efficiency of predators in catching prey, or the ability of prey to avoid predators.^63^ Given that night temperature is increasing faster than day temperature, nocturnal species may be especially vulnerable to interactive effects between light and temperature.^64^ Moreover, rising day temperature may increasingly shift human activities to the night, which may further increase light pollution.

Interactions with noise have been recorded for birds, including synergistic, antagonistic, and emergent effects, but effects on other taxa are poorly known.^65^ Both light and noise can stress individuals and influence their behavior, as well as alter their phenology and timing of daily activities.^66^ For instance, some birds initiate their dawn singing earlier in the morning under light pollution to avoid the interference from traffic noise.^67^

Interactions with chemical pollution is poorly known but likely to be common, as both light and chemical pollution influence biological rhythms and activities. Moreover, the impact of chemicals often depends on biological rhythms, such as the circadian rhythm or the stage of the reproductive cycle, which in turn are themselves sensitive to light pollution, indicating that the two factors can amplify each other.^68^

Finally, light pollution can facilitate the invasion of alien species into urban environments, including pests and disease vectors, by stressing native species and reducing their competitive ability.^4^ Invasive species are often generalists and could be more successful at adjusting to light pollution than many native species.^47^ Once in urban habitats, these alien species could interact with light pollution in the impact on native species, further facilitating their invasion success.

## Gaps in our knowledge and future research avenues

While an increasing number of studies show that light pollution influences the physiology and behavior of organisms, impacts on wildlife in urban environments, where many other stressors abound, are still poorly known. Yet, considering the rapid growth of urban areas, and the importance of biodiversity for functional and resilient urban ecosystems, the topic needs more attention.

In particular, little is known about the mechanisms and pathways behind the impacts of light pollution on urban wildlife. Why species differ in sensitivity, and the relative contribution of exaptations, plastic adjustments, and genetic adaptation to persistence under light pollution are largely unexplored topics.^69^ In particular, the role of epigenetic modifications in facilitating, or possibly hampering, persistence would deserve more attention, especially as the modifications can be faster than genetic changes.^70^ To differentiate between these processes and their interactions, robust experimental designs are needed that consider the impact of confounding factors (e.g., through common garden experiments^71^) and dependencies among traits, and that include enough replicates for drawing reliable conclusions.^45^

Research on the consequences of responses to light pollution needs to progress from the study of individual species to consequences for urban biodiversity and community composition.^15^ Species are part of complex webs of interactions and their responses depend on how other species in the community are responding, including feedback loops and time lags.^34^

Changes in the web of species interactions can, in turn, scale up to influence ecosystem processes, such as nutrient cycling, biomass production, pollination, seed dispersal, air and water purification, and the suppression of pest species. To develop efficient mitigation strategies to reduce negative effects of light pollution on these functions, we need more information on how light pollution influences the ecological function of species. Such knowledge would contribute to ensure well-functioning and resilient urban ecosystems, and thereby also maintain the ecosystem services that we humans depend on, such as clean air and water, green spaces for recreation, urban heat regulation, and the control of pests.

Overall, research needs to expand to include a wider diversity of taxa and geographical areas. Current research is biased toward birds and flying insects in developed countries although urbanization is most rapidly expanding in developing countries, which also support much of the earth’s biodiversity.

The development of new technologies has facilitated the investigation of animal responses to environmental changes, but is awaiting wider adoption in urban light pollution research. The use of light sensors, automatic recording of species distributions and behavior, and machine learning to analyze data could significantly advance both the documenting of changes and the investigation of underlying processes.^72^^,^^73^

Furthermore, more attention needs to be directed to interactions among multiple anthropogenic stressors, given that urban areas differ in many aspects from natural environments. Nonlinear effects, feedback loops, time lags, and the spatiotemporal context and life history of species need to be considered, as well as the possibility that stressors induce contrasting selection pressures that can constrain adaptation.

Finally the measurement of light pollution needs to be standardized, as different approaches are applied within ecological research and across disciplines.^74^ Current approaches vary from single-point to full light field (full-sphere) measurements, while units used vary from light intensities based on human vision, lux (lx), to full-spectrum irradiance (or radiance) measurements presented as energy (W/m^2^) or photon flux (photons/m^2^/s) per area.

## References

[1] Gaston K.J., Davies T.W., Nedelec S.L., Holt L.A. (2017). Impacts of artificial light at night on biological timings. Annu. Rev. Ecol. Evol. Syst..

[2] Gaston K.J., Sánchez de Miguel A. (2022). Environmental impacts of artificial light at night. Annu. Rev. Environ. Resour..

[3] Knop E., Zoller L., Ryser R., Gerpe C., Hörler M., Fontaine C. (2017). Artificial light at night as a new threat to pollination. Nature.

[4] Coetzee B.W.T., Gaston K.J., Koekemoer L.L., Kruger T., Riddin M.A., Smit I.P.J. (2022). Artificial light as a modulator of mosquito-borne disease risk. Front. Ecol. Evol..

[5] Sánchez de Miguel A., Bennie J., Rosenfeld E., Dzurjak S., Gaston K.J. (2021). First estimation of global trends in nocturnal power emissions reveals acceleration of light pollution. Rem. Sens..

[6] Gao J., O'Neill B.C. (2020). Mapping global urban land for the 21st century with data-driven simulations and Shared Socioeconomic Pathways. Nat. Commun..

[7] Ahmad S.B., Ali A., Bilal M., Rashid S.M., Wani A.B., Bhat R.R., Rehman M.U. (2023). Melatonin and health: Insights of melatonin action, biological functions, and associated disorders. Cell. Mol. Neurobiol..

[8] Dominoni D.M., Borniger J.C., Nelson R.J. (2016). Light at night, clocks and health: from humans to wild organisms. Biol. Lett..

[9] Cox D.T.C., Sánchez de Miguel A., Bennie J., Dzurjak S.A., Gaston K.J. (2022). Majority of artificially lit Earth surface associated with the non-urban population. Sci. Total Environ..

[10] Kyba C.C.M., Altıntaş Y.Ö., Walker C.E., Newhouse M. (2023). Citizen scientists report global rapid reductions in the visibility of stars from 2011 to 2022. Science.

[11] Jechow A., Kyba C.C., Hölker F. (2020). Mapping the brightness and color of urban to rural skyglow with all-sky photometry. J. Quant. Spectrosc. Radiat. Transf..

[12] Tong J.C.K., Lau E.S.L., Hui M.C.Y., Kwong E., White M.E., Lau A.P.S. (2022). Light pollution spatial impact assessment in Hong Kong: Measurement and numerical modelling on commercial lights at street level. Sci. Total Environ..

[13] Smyth T.J., Wright A.E., Edwards-Jones A., McKee D., Queirós A., Rendon O., Tidau S., Davies T.W. (2022). Disruption of marine habitats by artificial light at night from global coastal megacities. Elementa-Science of the Anthropocene.

[14] Sánchez de Miguel A., Bennie J., Rosenfeld E., Dzurjak S., Gaston K.J. (2022). Environmental risks from artificial nighttime lighting widespread and increasing across Europe. Sci. Adv..

[15] Sanders D., Frago E., Kehoe R., Patterson C., Gaston K.J. (2021). A meta-analysis of biological impacts of artificial light at night. Nat. Ecol. Evol..

[16] Fonken L.K., Nelson R.J. (2014). The effects of light at night on circadian clocks and metabolism. Endocr. Rev..

[17] van Grunsven R.H.A., Donners M., Boekee K., Tichelaar I., van Geffen K.G., Groenendijk D., Berendse F., Veenendaal E.M. (2014). Spectral composition of light sources and insect phototaxis, with an evaluation of existing spectral response models. J. Insect Conserv..

[18] Inger R., Bennie J., Davies T.W., Gaston K.J. (2014). potential biological and ecological effects of flickering artificial light. PLoS One.

[19] Horváth G., Kriska G., Malik P., Robertson B. (2009). Polarized light pollution: a new kind of ecological photopollution. Front. Ecol. Env..

[20] Candolin U. (2019). Mate choice in a changing world. Biol. Rev..

[21] Wong B.B.M., Candolin U. (2015). Behavioral responses to changing environments. Behav. Ecol..

[22] Falcón J., Torriglia A., Attia D., Viénot F., Gronfier C., Behar-Cohen F., Martinsons C., Hicks D. (2020). Exposure to Artificial Light at Night and the Consequences for Flora, Fauna, and Ecosystems. Front. Neurosci..

[23] Angelier F., Wingfield J.C. (2013). Importance of the glucocorticoid stress response in a changing world: Theory, hypotheses and perspectives. Gen. Comp. Endocrinol..

[24] Both C., van Asch M., Bijlsma R.G., van den Burg A.B., Visser M.E. (2009). Climate change and unequal phenological changes across four trophic levels: constraints or adaptations?. J. Anim. Ecol..

[25] Bennie J., Davies T.W., Cruse D., Inger R., Gaston K.J. (2018). Artificial light at night causes top-down and bottom-up trophic effects on invertebrate populations. J. Appl. Ecol..

[26] Ganguly A., Candolin U. (2023). Impact of light pollution on aquatic invertebrates: Behavioral responses and ecological consequences. Behav. Ecol. Sociobiol..

[27] Arlettaz R., Godat S., Meyer H. (2000). Competition for food by expanding pipistrelle bat populations (*Pipistrellus pipistrellus*) might contribute to the decline of lesser horseshoe bats (*Rhinolophus hipposideros*). Biol. Conserv..

[28] Levy K., Fishman B., Barnea A., Ayali A., Tauber E. (2022). Transcriptional response of circadian clock genes to an 'Artificial Light at Night' pulse in the cricket *Gryllus bimaculatus*. Int. J. Mol. Sci..

[29] Hui C.K., Chen N., Chakraborty A., Alaasam V., Pieraut S., Ouyang J.Q. (2023). Dim artificial light at night alters immediate early gene expression throughout the avian brain. Front. Neurosci..

[30] Touzot M., Lefebure T., Lengagne T., Secondi J., Dumet A., Konecny-Dupre L., Veber P., Navratil V., Duchamp C., Mondy N. (2022). Transcriptome-wide deregulation of gene expression by artificial light at night in tadpoles of common toads. Sci. Total Environ..

[31] He Y., Ganguly A., Lindgren S., Quispe L., Suvanto C., Zhao K., Candolin U. (2024). Carry-over effect of artificial light at night on daytime mating activity in an ecologically important detritivore, the amphipod Gammarus pulex. J. Exp. Biol..

[32] Gaston K.J., Bennie J. (2014). Demographic effects of artificial nighttime lighting on animal populations. Environ. Rev..

[33] Knell R.J. (2009). Population density and the evolution of male aggression. J. Zool..

[34] Hirt M.R., Evans D.M., Miller C.R., Ryser R. (2023). Light pollution in complex ecological systems. Phil. Trans. R. Soc. B..

[35] Hopkins G.R., Gaston K.J., Visser M.E., Elgar M.A., Jones T.M. (2018). Artificial light at night as a driver of evolution across urban-rural landscapes. Front. Ecol. Env..

[36] Tuomainen U., Candolin U. (2011). Behavioural responses to human-induced environmental change. Biol. Rev..

[37] Kolláth Z., Jechow A. (2023). Natural variation of the colour and spectrum of the night sky observed at a potential european reference site for dark skies. J. Quant. Spectrosc. Radiat. Transf..

[38] Bell G. (2017). Evolutionary Rescue. Annu. Rev. Ecol. Evol. Syst..

[39] Gomes E., Rey B., Débias F., Amat I., Desouhant E. (2021). Dealing with host and food searching in a diurnal parasitoid: consequences of light at night at intra- and trans-generational levels. Insect Conserv. Divers..

[40] McGuigan K., Hoffmann A.A., Sgrò C.M. (2021). How is epigenetics predicted to contribute to climate change adaptation? What evidence do we need?. Phil. Trans. R. Soc. B..

[41] Danchin E., Giraldeau L.A., Valone T.J., Wagner R.H. (2004). Public information: From nosy neighbors to cultural evolution. Science.

[42] Des Roches S., Brans K.I., Lambert M.R., Rivkin L.R., Savage A.M., Schell C.J., Correa C., De Meester L., Diamond S.E., Grimm N.B. (2021). Socio-eco-evolutionary dynamics in cities. Evol. Appl..

[43] Alberti M., Correa C., Marzluff J.M., Hendry A.P., Palkovacs E.P., Gotanda K.M., Hunt V.M., Apgar T.M., Zhou Y. (2017). Global urban signatures of phenotypic change in animal and plant populations. Proc. Natl. Acad. Sci. USA.

[44] Johnson M.T.J., Munshi-South J. (2017). Evolution of life in urban environments. Science.

[45] Lambert M.R., Brans K.I., Des Roches S., Donihue C.M., Diamond S.E. (2021). Adaptive Evolution in Cities: Progress and Misconceptions. Trends Ecol. Evol..

[46] Sol D., Lapiedra O., Ducatez S., Szulkin M., MunshiSouth J., Charmantier A. (2020). Urban Evolutionary Biology.

[47] Komine H., Koike S., Schwarzkopf L. (2020). Impacts of artificial light on food intake in invasive toads. Sci. Rep..

[48] Stone E.L., Jones G., Harris S. (2009). Street Lighting Disturbs Commuting Bats. Curr. Biol..

[49] Lao S., Robertson B.A., Anderson A.W., Blair R.B., Eckles J.W., Turner R.J., Loss S.R. (2020). The influence of artificial light at night and polarized light on bird-building collisions. Biol. Conserv..

[50] van Langevelde F., Braamburg-Annegarn M., Huigens M.E., Groendijk R., Poitevin O., van Deijk J.R., Ellis W.N., van Grunsven R.H.A., de Vos R., Vos R.A. (2018). Declines in moth populations stress the need for conserving dark nights. Glob. Chang. Biol..

[51] Kamrowski R.L., Limpus C., Moloney J., Hamann M. (2012). Coastal light pollution and marine turtles: assessing the magnitude of the problem. Endanger. Species Res..

[52] Elgert C., Hopkins J., Kaitala A., Candolin U. (2020). Reproduction under light pollution: maladaptive response to spatial variation in artificial light in a glow-worm. Proc. Biol. Sci..

[53] Kivelä L., Elgert C., Lehtonen T.K., Candolin U. (2023). The color of artificial light affects mate attraction in the common glow-worm. Sci. Total Environ..

[54] Sanders D., Gaston K.J. (2018). How ecological communities respond to artificial light at night. J. Exp. Zool. A Ecol. Integr. Physiol..

[55] Altermatt F., Ebert D. (2016). Reduced flight-to-light behaviour of moth populations exposed to long-term urban light pollution. Biol. Lett..

[56] Jones T.M., Llamas A.P., Phillips J.N. (2023). Phenotypic signatures of urbanization? Resident, but not migratory, songbird eye size varies with urban-associated light pollution levels. Glob. Chang. Biol..

[57] Schmidt C., Domaratzki M., Kinnunen R.P., Bowman J., Garroway C.J. (2020). Continent-wide effects of urbanization on bird and mammal genetic diversity. Proc. Biol. Sci..

[58] Miles L.S., Rivkin L.R., Johnson M.T.J., Munshi-South J., Verrelli B.C. (2019). Gene flow and genetic drift in urban environments. Mol. Ecol..

[59] Halfwerk W. (2021). How Should We Study Urban Speciation?. Front. Ecol. Evol..

[60] Grimm N.B., Faeth S.H., Golubiewski N.E., Redman C.L., Wu J., Bai X., Briggs J.M. (2008). Global change and the ecology of cities. Science.

[61] Alberti M., Wang T. (2022). Detecting patterns of vertebrate biodiversity across the multidimensional urban landscape. Ecol. Lett..

[62] Dominoni D.M., Kjellberg Jensen J., de Jong M., Visser M.E., Spoelstra K. (2020). Artificial light at night, in interaction with spring temperature, modulates timing of reproduction in a passerine bird. Ecol. Appl..

[63] Miller C.R., Barton B.T., Zhu L., Radeloff V.C., Oliver K.M., Harmon J.P., Ives A.R. (2017). Combined effects of night warming and light pollution on predator-prey interactions. Proc. Biol. Sci..

[64] Gaston K.J., Gardner A.S., Cox D.T.C. (2023). Anthropogenic changes to the nighttime environment. Bioscience.

[65] Halfwerk W., Jerem P. (2021). A systematic review of research investigating the combined ecological impact of anthropogenic noise and artificial light at night. Front. Ecol. Evol..

[66] McMahon T.A., Rohr J.R., Bernal X.E. (2017). Light and noise pollution interact to disrupt interspecific interactions. Ecology.

[67] Da Silva A., Valcu M., Kempenaers B. (2016). Behavioural plasticity in the onset of dawn song under intermittent experimental night lighting. Anim. Behav..

[68] Zheng X., Zhang K., Zhao Y., Fent K. (2021). Environmental chemicals affect circadian rhythms: An underexplored effect influencing health and fitness in animals and humans. Environ. Int..

[69] Verrelli B.C., Alberti M., Des Roches S., Harris N.C., Hendry A.P., Johnson M.T.J., Savage A.M., Charmantier A., Gotanda K.M., Govaert L. (2022). A global horizon scan for urban evolutionary ecology. Trends Ecol. Evol..

[70] Boskovic A., Rando O.J., Bonini N.M. (2018).

[71] Czaczkes T.J., Bastidas-Urrutia A.M., Ghislandi P., Tuni C. (2018). Reduced light avoidance in spiders from populations in light-polluted urban environments. Sci. Nat..

[72] Couzin I.D., Heins C. (2023). Emerging technologies for behavioral research in changing environments. Trends Ecol. Evol..

[73] Besson M., Alison J., Bjerge K., Gorochowski T.E., Høye T.T., Jucker T., Mann H.M.R., Clements C.F. (2022). Towards the fully automated monitoring of ecological communities. Ecol. Lett..

[74] Hölker F., Bolliger J., Davies T.W., Giavi S., Jechow A., Kalinkat G., Longcore T., Spoelstra K., Tidau S., Visser M.E., Knop E. (2021). 11 pressing research questions on how light pollution affects biodiversity. Front. Ecol. Evol..

